# Stable Reusability of Nanocellulose Aerogels with Amino Group Modification in Adsorption/Desorption Cycles for CO_2_ Capture

**DOI:** 10.3390/ma18020243

**Published:** 2025-01-08

**Authors:** Fabiola Valdebenito, Camila Albornoz, Valentina Rivera, Elizabeth Elgueta, Muhammad Nisar, Sebastian Lira, Oscar Valerio, Ana Narváez, Carolina Quezada, Robinson Muñoz, Laura Azócar, Franco Sandoval

**Affiliations:** 1 Departamento de Química Ambiental, Universidad Católica de la Santísima Concepción, Concepción 4070129, Chile; calbornoz@qaciencias.ucsc.cl (C.A.); vrivera@qaciencias.ucsc.cl (V.R.); eelgueta@ucsc.cl (E.E.); carolina.quezada@ucsc.cl (C.Q.); robinson.munoz@ucsc.cl (R.M.); lazocar@ucsc.cl (L.A.); fsandoval395@gmail.com (F.S.); 2 Centro de Energía, Universidad Católica de la Santísima Concepción, Concepción 4070129, Chile; mnisar@ucsc.cl (M.N.); anarvaez@ucsc.cl (A.N.); 3 Departamento de Ingeniería Eléctrica, Universidad Católica de la Santísima Concepción, Concepción 4070129, Chile; 4 Center for Sustainability Research, Universidad Andres Bello, Santiago 7591538, Chile; sebalira@gmail.com; 5 Departamento de Ingeniería Química, Universidad de Concepción, Concepción 4030000, Chile; oscarvalerio@udec.cl; 6 Centro de Investigación de Polímeros Avanzados, CIPA, Concepción 4051381, Chile

**Keywords:** nanocellulose aerogels, CO_2_ capture, amino load

## Abstract

This study evaluated the stability and reusability of amino-functionalized nanocellulose aerogels as CO_2_-adsorbent materials. The modified aerogels, synthesized via a controlled silylation using N-[3-(trimethoxysilyl) propyl] ethylenediamine (DAMO), demonstrated excellent thermal stability up to 250 °C (TGA) and efficient CO_2_ adsorption through chemisorption, which was the main adsorption mechanism. The performance of the aerogels was assessed using both adsorption isotherms and the decay pressure technique, revealing that CO_2_ adsorption capacity increased with higher amino group loading (4.62, 9.24, and 13.87 mmol of DAMO). At 298 K and 4 bar, CO_2_ adsorption capacity increased proportionally with the amino group concentration, reaching values of 3.17, 5.98, and 7.86 mmol of CO_2_ g^−1^ polymer, respectively. Furthermore, over 20 adsorption/desorption cycles, the aerogels maintained 95% CO_2_ desorption at ambient temperature, indicating their potential for industrial use. These findings highlight the aerogels suitability as stable, reusable materials for large scale CO_2_ capture and storage technologies.

## 1. Introduction

CO_2_ is considered the most important greenhouse gas, due to the continuous increase in atmospheric CO_2_ concentration from 310 ppm in 1960 to 410 ppm in 2019 [1]. To reduce CO_2_ concentrations, it is necessary to implement measures such as: reducing deforestation, promoting the use of more efficient energy, using renewable energy sources, and applying carbon capture and storage (CCS) as well as capture and storage utilization (CCU) technologies [2]. The main carbon capture technologies are based on absorption and adsorption operations, membrane separation, and cryogenic distillation technologies, the first two being the most studied [3].

Adsorption technologies using solid porous materials seems to be an efficient alternative for CO_2_ capture due to its reusable nature, low cost, versatility, and easy operations. The literature reports on different adsorbent materials with high selectivity, including amine-modified silica, amines supported on porous carbon, and/or on other metal oxides such as alumina, zeolites, metal oxides, and metal–organic frameworks (MOFs) [4,5]. The development of some of these materials is limited due to drawbacks such as their hydrophilic nature, which requires a drying step prior to use. Therefore, the introduction of hydrophobic sites has been considered to overcome this limitation. In fact, hydrophobic microporous solids are more resistant to the presence of water vapor but tend to absorb less CO_2_ [4,6]. Furthermore, the high temperatures required for the regeneration of these materials after being used for CO_2_ adsorption is also a major obstacle [4,6]. To address the limitations presented by inorganic absorbents, during the last decade, the use of bio-based absorbents for CO_2_ capture has been extensively investigated, emphasizing cellulose nanofibril (CNF) [7,8].

The chemical modification of CNF for CO_2_ capture using silane coupling agents is considerably investigated [7,8,9,10,11,12,13]. Gebald et al. (2014) was the first to suggest the use of nanocelluloses as a solid support for CO_2_ adsorption, achieving an adsorption of 1.39 mmol CO_2_ g^−1^ [14].

Similarly, nanocellulose adsorbents were modified using aminosilanes, namely N-(2-aminoethyl)-3-aminopropylmethyldimethoxysilane, phthalimide (1,3-dihydro-1,3-dioxoisoindole), N-(2-aminoethyl)-3-aminopropylmethyldimethoxysilane, (3-trimethoxysilylpropyl) diethylenediamine, and N-(2-aminoethyl)-3-aminopropylmethyldimethoxysilane (AEAPDMS), achieving a CO_2_ adsorption of between 0.5 and 5 mmol CO_2_ g^−1^ [7,8,9,10,11,12,13]. Valdebenito et al. (2018) reported the synthesis of nanocellulose thin films for CO_2_ adsorption from corn husks, oat hulls, and kraft pulp, and modified them using 3-aminopropyltrimethoxysilane, N-(2-aminoethyl)-3-aminopropyltrimethoxysilane, [3-(trimethoxysilyl) propyl] ethylenediamine, and (3-trimethoxysilylpropyl) diethylenetriamine. The nanocellulose film derived from kraft pulp, modified using [3-(trimethoxysilyl) propyl] ethylenediamine, had the highest CO_2_ adsorption capacity of 2.11 mmol g^−1^ at room temperature and atmospheric pressure. It is worth noting that this modified nanocellulose thin film has the highest amine content, but the lowest surface area, demonstrating that chemisorption was the dominant adsorption type [8].

Due to the significant effect of amino loading on CO_2_ adsorption capacity, it would be valuable to further explore how this variable affects the maximum CO_2_ reversible adsorption capacity of the aerogel (CO_2_ adsorption and desorption stages) and its lifespan.

The main objective of this study was to evaluate the maximum reversible CO_2_ adsorption capacity, stability, and lifespan of amino-functionalized nanocellulose aerogels as CO_2_ adsorbent materials. By using the decay pressure technique, which allows measurements at pressures higher than atmospheric, to assess the effect of pressure on CO_2_ adsorption, this approach provides valuable insights into the potential of these materials for long-term use in CCS and CCU technologies.

## 2. Materials and Methods

The nanocellulose hydrogels were obtained from commercial cellulose (kraft pulp), N-[3-(Trimethoxysilyl) propyl] ethylenediamine (DAMO), (2,2,6,6,6-tetramethylpiperidinyl-1-oxyl) TEMPO, NaClO, NaClO_2_, NaOH, glacial acetic acid, and ethanol, which were purchased from Merck/Sigma Aldrich, Santiago de, Chile.

### 2.1. Synthesis of Nanocellulose Aerogels

The TEMPO-mediated oxidation of cellulose suspension was carried out according to Saito et al. (2005) with some modifications [15]. The oxidized fibers were dispersed in deionized water with a pulp concentration of 1 wt.% and then homogenized with a high-pressure homogenizer (NS1001L PANDA 2K-GEA) 8 times at a pressure drop of 800 bar to produce nanocellulose hydrogels. To produce nanocellulose aerogels from hydrogels, the freeze-dried technique of an aqueous suspension of nanocellulose was used. This suspension was poured into a mold, then the samples were frozen at –21 °C for at least 24 h to be lyophilized (FreeZone 6, Labconco) at –41 °C and 0.01 mbar for 12–30 h depending on the amount of water to remove. At the end of this process, nanocellulose aerogels were obtained.

### 2.2. Nanocellulose Aerogel Silylation with N-[3-(Trimethoxysilyl) Propyl] Ethylenediamine (DAMO)

**Figure 1:**
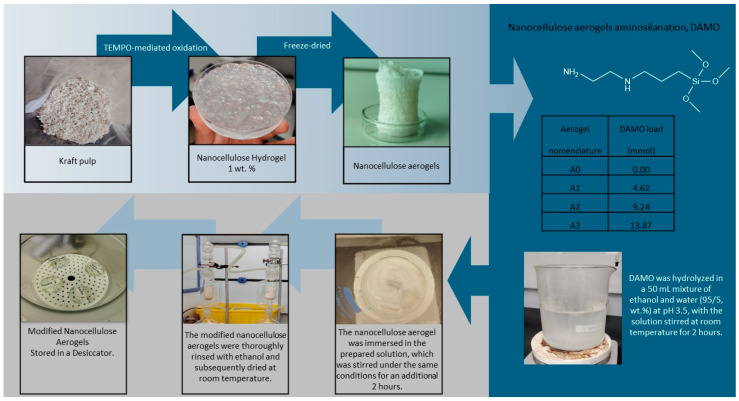
Reaction scheme preparation of amino-modified nanocellulose aerogels.

The obtained aerogels were functionalized with 3 different amino group loads, i.e., 4.62, 9.24, and 13.87 mmol portions of DAMO (see Figure 1), which were hydrolyzed in a 50 mL mixture of ethanol/water (95/5, wt.%). The pH was adjusted to 3.5 by adding acetic acid, and the solution was stirred at room temperature for 2 h. Then, nanocellulose aerogel was immersed into this solution. The solution was stirred for 2 h at room temperature. This protocol was repeated for the 3 amino group loadings. The modified nanocellulose aerogels were thoroughly washed with ethanol (soxhlet extraction with ethanol) before being dried at room temperature in a closed system and stored in a desiccator to avoid humidity [8].

### 2.3. Characterization

The analysis of functional groups in the nanocellulose aerogel was carried out through infrared spectroscopy. The IR measurements were performed with an Agilent Tensor 27 instrument (Malaysia) in Fourier transform mode (FTIR). The Agilent MicroLab PC software (version IQ/OQ, 21 CFR Part 11 compliant) was used for data acquisition. A total of 40 scans were collected across a spectral range of 400 to 4000 cm^−1^ [7,8].

The carbon, hydrogen, and nitrogen contents in the nanocellulose aerogels were quantified using an EA 3000 Elemental Analyzer, from Perkin Elmer, Chile. The analysis was performed in triplicate for each sample. The samples were weighed on a microbalance (Sartorius CP2-P) between 1.5 and 2.5 mg for each sample. Each sample was subsequently placed in a previously tared tin capsule. The elemental analyzer was calibrated using 5-level L-cysteine (0.1, 0.25, 0.5, 1.0, and 2.0 mg), and the encapsulated sample was loaded into the equipment. The 21 CFR software (version Part 11) was used for data acquisition. 

Thermo-gravimetric analysis was performed using a STA 6000 apparatus from Perkin Elmer Chile, at a heating rate of 10 °C min^−1^ up to 800 °C under the nitrogen atmosphere (flow rate = 90 mL min^−1^). About 30 mg of each sample were analyzed. The Pyris software (version 13.4.0.0036) was used for data acquisition [7,8,13].

Scanning electron microscopy (VP-SEM) analysis was performed. The samples were adhered to the sample holder with double-sided carbon tape. The sample was visualized using a backscatter detector (BSE) in variable pressure without any further sputtering under the following parameters: 10 KV energy, 20 Pa pressure, and WD 10 mm in a scanning electron microscope (Hitachi SU3500, Tokyo, Japan). The images were acquired and analyzed with Hitachi software controller and Image J 1.53k Java 1.8.0_172 Software (Wayne Rasband and contributors, National Institutes of Health, Betheda, MA, USA) [8,9,13].

For the X-ray photoelectron spectroscopy (XPS) analysis, an Axis Ultra DLD electron spectrometer was used. Survey scans were recorded using monochromated Al Kα irradiation with a 50 W, 0.1 eV step, and 160 eV analyzer pass energy. Analysis = 700 um × 300 um; charge neutralizer, ON; narrows scans (elemental quantification and peak fitting), pass energy = 20 eV; energy calibration, C 1s C-(C, H) component @285.0 eV [7,8].

### 2.4. CO_2_ Adsorption Measurements

The CO_2_ adsorption was measured through CO_2_ adsorption isotherms at 273 K (relative pressure: 0.00002–0.02) using Micromeritis Tristar II 3020 equipment, with samples degassed at room temperature for 48 h. The adsorption isotherms were adjusted using the BET, Langmuir, Freundlich, and Temkin models (see Table 1) [7,8,13].

The pressure decay technique was used to determine CO_2_ adsorption capacity. The dual-chamber gas sorption cell followed the method of Koros et al. (1976). Before measurements, 0.7 g^−1^ g of the sample were weighed and dried for 1 h at 70 °C (343 K). CO_2_ sorption experiments were carried out at 25 °C (298 K) and at 4 bar. CO_2_ sorption capacity was calculated using Equations (1) and (2) [16].

(1) $$n_{CO_{2}}=\frac{P_{i}V_{gc}}{Z_{\left(P_{i},T_{i}\right)}RT_{i}}-\frac{P_{eq}\left(V_{t}-V_{p}\right)}{Z_{\left(P_{eq},T_{eq}\right)}RT_{eq}}$$

(2) $$w_{CO_{2}/g}=\frac{n_{CO_{2}}M}{W_{s}}$$

Here, *wCO*_2_*/g* is the weight of gas adsorbed by the sample, *P_i_* and *T_i_* give the pressure and the temperature in the gas chamber, respectively, and those parameters at equilibrium are represented as *P_eq_* and *T_eq_*; *V_gc_* is the gas chamber’s volume, *V_p_* is the volume of the sample, and *V_t_* is the total volume of the sorption cell. The coefficient of compressibility “*Z*” for CO_2_ was obtained via the Span–Wagner equations of state [16,17].

### 2.5. Nanocellulose Aerogel Adsorption/Desorption Cycle Study (TGA)

Adsorption/desorption cycle analysis was performed on a STA 6000 apparatus from Perkin Elmer; it used a flow of 50 mL min^−1^ of N_2_ (99.999% purity) for 24 h for sample degassing. Then, a 20 mL min^−1^ of CO_2_ flow (99.999% purity) was added and was maintained for 30 min. Subsequently, N_2_ conditions were resumed for 30 min. The analysis was carried out at room temperature 298 K 20 times.

## 3. Results

### 3.1. Infrared Spectroscopy Analysis (FTIR)

**Figure 2:**
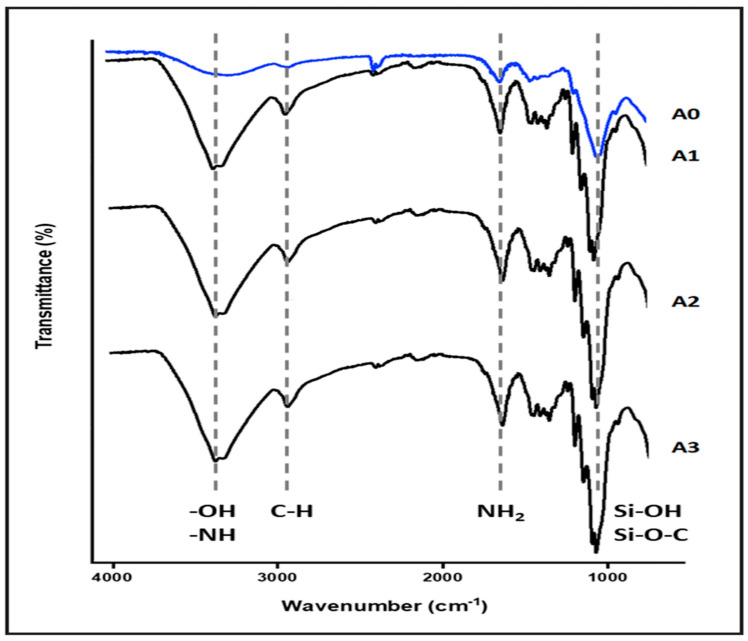
Nanocellulose aerogel FTIR spectra.

The FTIR spectra of both nanocellulose aerogel unmodified and modified with di-aminosilane are shown in Figure 2. The spectrum of unmodified aerogel (A0) exhibited typical bands for cellulose, such as O-H stretching at 3200 cm^−1^, C-H stretching at 2900 cm^−1^, CH_2_ symmetric bending at 1400 cm^−1^, and O-H and C-H bending as well as C-C and C-O stretching at 1380, 1310, and 1250 cm^−1^ respectively.

The spectrum of aerogel modified (A1, A2, and A3) showed the successful grafting of di-amino silane on nanocellulose. The band at 2900 cm^−1^ was assigned to C-H stretching, the O-H stretching at 3200 cm^−1^ was replaced for the band at 3300 cm^−1^ assigned to N-H stretching. Also, the band at 1700 cm^−1^ assigned to N-H flexion and the appearance of signals associated with vibrations from silicon-based linkages were observed at 1240 cm^−1^, and between 1180 and 700 cm^−1^ (Si-OH, Si-O-C) [8,9,13,14].

### 3.2. Elemental Analysis (C, H, and N)

The elemental distribution over the surface was confirmed by elemental analysis (C, H, and N) and are shown in Table 2. The constituents C, O, and N show that the grafting of the amino group took place in the polymer. From Table 2, it can be observed that increasing the amino group content in the aerogel led to an increase in the nitrogen percentage. Interestingly, a linear relationship was observed between the amino group content and nitrogen content due to the grafting of amino groups onto the nanocellulose matrix.

### 3.3. Nanocellulose Aerogel Thermo-Gravimetric Analysis (TGA)

**Figure 3:**
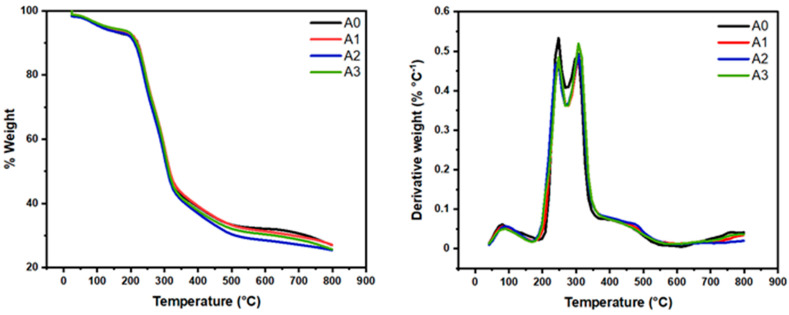
TGA/DTG curves of nanocellulose aerogels.

The functionalized aerogels (A1, A2, and A3) were compared to unmodified A0. The weight loss and derivative of weight loss versus temperature plots (Figure 3) for A0 show multiple degradation events. The onset of thermal degradation is practically the same for A0 and A1 aerogels.

All sample thermograms demonstrate two weight loss events preceding the main decomposition step, as can be seen from Figure 3. The first degradation (at between 90 and 100 °C) may be attributed to loss of some bound volatile material, probably residual water. Similarly, the peak around 240 °C in all aerogel’s samples, correspond to hemicelluloses that were not removed during the pulping process. Finally, the peak around 320° in all samples corresponds to the degradation temperatures of the cellulose [7,8,9,14]. The developed nanomaterials (biodegradable materials) exhibited adequate thermal stability up to 240 °C, which is one of the pre-requisites of the CO_2_ capture materials at the industrial scale. 

### 3.4. Scanning Electron Microscopy (VP-SEM)

**Figure 4:**
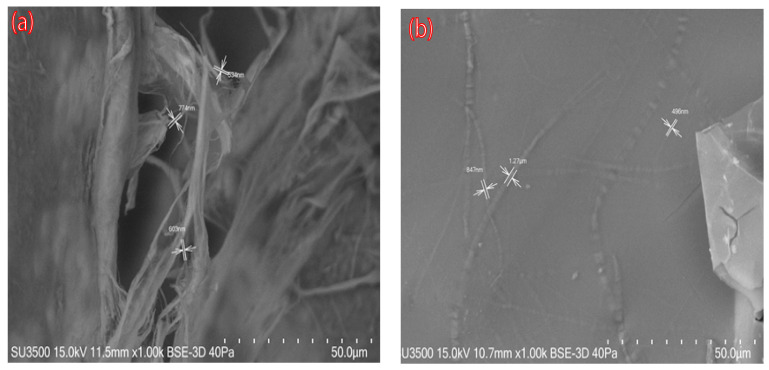
SEM images of nanocellulose aerogels: (**a**) A0, unmodified nanocellulose aerogel; (**b**) A1, DAMO-grafted nanocellulose aerogel.

SEM images of nanocellulose aerogels modified with DAMO A0 and A1 (A1; like A2 and A3 images) are shown in Figure 4. The unmodified aerogel exhibited a random pore structure and disorderly cross-linked CNFs were observed. After amino group grafting, many planar structures with individual CNFs irregularly attached to the cellulose sheet were observed (Figure 4b). These results are in accordance with previously reported studies in the literature [9,13].

### 3.5. X-Ray Photoelectron Spectroscopy (XPS)

**Figure 5:**
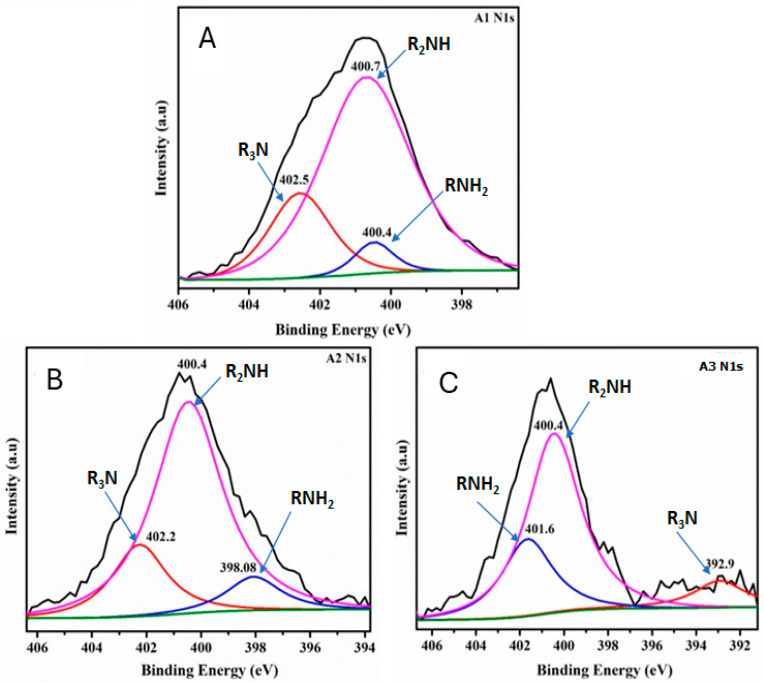
N1s XPS deconvolution curves for samples A1 (**A**), A2 (**B**), and A3 (**C**). The pink, red, and blue curves represent the R_2_NH, R_3_N, and RNH_2_ groups, respectively, indicating the anchoring of nitrogen-containing functional groups onto the aerogel samples. The black line corresponds to baseline noise, while the green line represents the baseline.

XPS measurements were next performed to further analyze the chemical composition and surface electronic state of the CNF aerogel samples (Figure 5). The binding energy (BE) peaks from the N1s at 398–402 eV appeared in the A1, A2, and A3 samples. The N1s spectrum of modified CNF aerogels (Figure 5A–C) can be fitted to three peaks with the BE at 400 (R_2_NH and RNH_2_) and 402.0 (R_3_N) eV, demonstrating that the DAMO was anchored to the surface of CNF aerogels [7,8,13].

The N1s deconvolution spectrum reveals that as the amino group loading in the aerogels increases (Figure 5A–C), the signal intensity for the R_3_N and R_2_NH groups decreases, while the intensity of the RNH_2_ group increases. Consequently, aerogel A3 exhibits the highest percentage of NH_2_ groups grafted onto the nanocellulosic matrix, making them available for CO_2_ capture.

Another finding observed in the N1s deconvolution spectra is that as the amino group content increases (Figure 5A–C), the peak corresponding to the RNH_2_ group shifts toward the higher binding energy region. This shift can be attributed to changes in local electronegativity caused by the formation of the carbamate ion. The formation of this ion alters the electronic density, resulting in the RNH_2_ peak moving to higher binding energies.

Additionally, this phenomenon may be influenced by polarization effects, which enhance the reactivity of the RNH_2_ group toward CO_2_. This increased reactivity contributes to a higher CO_2_ adsorption capacity in aerogels modified with a greater amino group content. Such behavior could be explained by the overlap of C-N atomic orbitals. In other words, changes in the amino group charge induce alterations in the electronic density of the covalent bond, facilitating CO_2_ chemisorption and improving the aerogel’s performance.

### 3.6. CO_2_ Absorption Isotherms at 273 K

**Figure 6:**
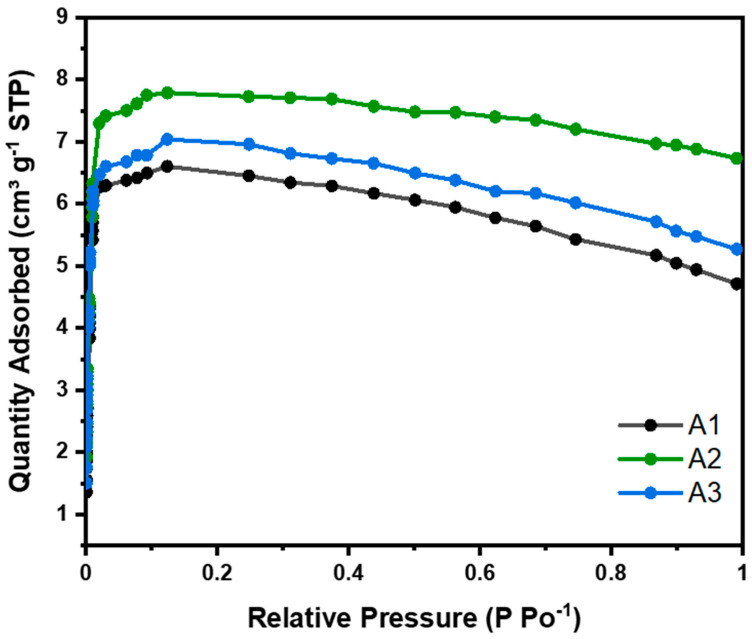
Nanocellulose aerogel CO_2_ adsorption isotherms at 273 K.

CO_2_ adsorption isotherms were carried out at 273 K for all samples studied and were classified as type 1 (see Figure 6) in the Brunauer–Deming–Deming–Teller (BDDT) isotherm classification, corresponding to microporous solids. This classification indicates monolayer adsorption, typical of microporous materials with strong adsorbate–adsorbent interactions. To calculate the CO_2_ adsorption capacity, the experimental data were fitted to Langmuir, BET, Freundlich, and Temkin models (see Supporting Information). The deviation from the typical type 1 isotherm behavior observed in Figure 6 at higher relative pressures, resulting from volume swelling, has been corrected in the linearized adsorption isotherms of CO_2_ (Langmuir and BET), as presented in the Supplementary Information (Supporting Information).

As the relative pressure increased, the CO_2_ adsorption capacity reached a maximum at approximately 0.4 bar (micropore filling) and subsequently decreased gradually. This phenomenon has also been reported in other studies and can be attributed to the nonlinearity of the apparent CO_2_ density with temperature and pressure [18,19].

Table 3 shows some parameters derived from the adsorption isotherms for nanocellulose aerogels at 273 K. Qm corresponds to the amount adsorbed in cm^3^ g^−1^, calculated for each linearized isotherm model under standard temperature and pressure conditions. Based on this parameter, the adsorbed amount Xo (mmol g^−1^) from Table 4 is determined using the ideal gas law equation. R is the correlation coefficient, indicating how well our experimental data fit each model. Based on these criteria, the isotherm classification (BDDT), and the reaction mechanism, the most suitable model is selected. The adsorption isotherms at 273 K are kept at low temperatures to measure the amount of CO_2_ that can be adsorbed by a material at different relative pressures. At these temperatures, the adsorption is physical, allowing for the evaluation of the material’s maximum adsorption capacity without the effects of chemical reactions at higher temperatures. This technique is very useful for characterizing porous materials and comparing their adsorption capacity under controlled conditions. However, the results obtained at these temperatures may not represent the material behaviors at temperatures more relevant to practical applications, such as CO_2_ capture in environmental or industrial conditions.

For physical adsorption in multilayers, the BET model was used to calculate the amount of CO_2_ adsorbed. Nanocellulose aerogels modified with different amino-silane loads, i.e., 4.62, 9.24, and 13.87 mmol, showed CO_2_ adsorptions of 0.21, 0.25, and 0.22 mmol CO_2_ g^−1^. At 273 K (see Table 4), this adsorption was due to physisorption. No significant differences were observed in the adsorption of the three aerogels studied. Aerogel A2 showed slightly higher CO_2_ adsorption, due to having a slightly larger surface area than the others, as can be seen in Table 4.

Baraka et al. (2024), indicate that most cellulose nanofiber aerogels reported in the literature have a BET surface area ranging from 7.1 to 335 m^2^ g^−1^. Therefore, our results are in close agreement (see Table 4, 154 m^2^ g^−1^) when obtained through the same drying method (freeze-drying) and functionalization method (liquid phase) [20].

### 3.7. CO_2_ Absorption Capacity Through Pressure Decay Technique at 298 K and 4 Bar

**Figure 7:**
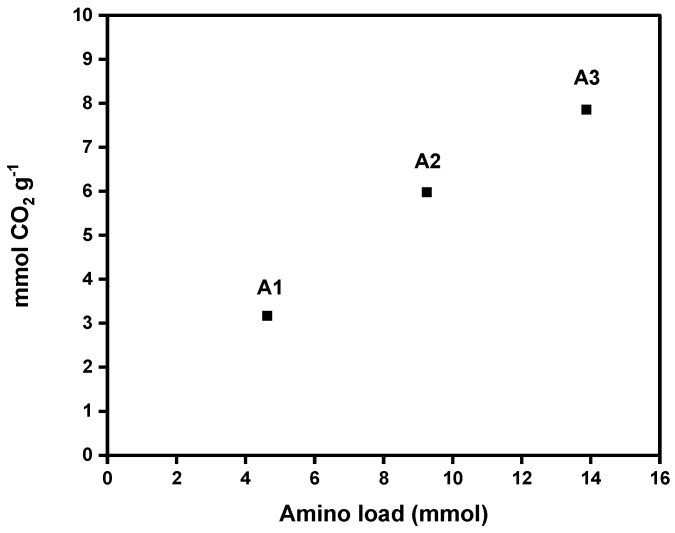
Amino group load effect on the CO_2_ adsorption capacity of nanocellulose aerogels calculated by the pressure decay technique.

Figure 7 shows CO_2_ adsorption capacities of each aerogel (A1, A2, and A3). The values obtained for the DAMO-modified nanocellulose aerogels ranged from 3.17 to 7.86 mmol CO_2_ g^−1^ at 25 °C, proving a significant increase in CO_2_ adsorption with an increase in amino group content in the nanocellulose aerogel. These results are promising. The literature reports CO_2_ adsorption capacities of amine-modified cellulose nanofiber (CNF) aerogels ranging from 1.39 to 2.4 mmol g^−1^ (Table 5). These values are lower than those achieved in the present work at the same adsorption temperature (see Table 5). Sepahvand et al. (2020) obtained 5.2 mmol g^−1^ of CO_2_ adsorption from CNF aerogels modified with phthalimide (1.5% phthalimide content) under the same freeze-drying and liquid phase functionalization methods, using a nonlinear amine (phthalimide). However, this value is lower than the adsorption capacities achieved by the samples A2 and A3, which attained CO_2_ capture values of 5.98 and 7.86 mmol g^−1^, respectively.

Moreover, it can be observed that the obtained results are higher than the adsorption capacities of other typical adsorbents used in CO_2_ capture. For example, the APTS-modified MCM-41 reached a value of 1.33 mmol CO_2_ g^−1^ [21]. Furthermore, MCM-41 exhibited a higher BET surface area (1602 m^2^ g^−1^) than the nanocellulose aerogels studied in the present work.

The high adsorption values obtained are mainly explained by the chemisorption mechanisms between the amino groups and CO_2_ molecules. The reaction of CO_2_ with DAMO involves a direct reaction between the carbon atom of CO_2_ and the nitrogen atom of the amine, forming a covalent bond [22,23]. Therefore, the chemical modification of nanocellulose with amine moieties enhances the adsorption capacity by providing many active binding sites that promote interactions between the aerogel and CO_2_ molecules [22,23]. The molar ratio of amine introduced could also significantly influence the CO_2_ adsorption efficiency of modified nanocellulose aerogels. A high concentration of amine promotes a high adsorption capacity [24].

This trend is consistent with the results obtained from the XPS analysis proving the higher % NH_2_ where a linear tendency was observed. With a higher amino group loading, there was an increased nitrogen content (amino group according to the supported amine reaction mechanism via carbamate ions) that was grafted onto the nanocellulosic matrix. Additionally, there was also greater CO_2_ adsorption capacity measured at room temperature. Regarding our results, a significant increase in CO_2_ adsorption is observed with a slight increase in DAMO loading (4.62, 9.24, and 13.87 mmol of DAMO, respectively). The pressure decay technique can be used at different temperatures and pressures, allowing for a simulation closer to real operating conditions. It is useful for studying the kinetics of adsorption and desorption and the adsorption capacity [20]. 

Unlike most studies reported in the literature, this research uses the pressure decay technique to determine the maximum reversible adsorption CO_2_ capacity of amino-functionalized CNF aerogel. This method allows for pressures above atmospheric levels, making it possible to evaluate how increased pressure affects the adsorbent capacity at room temperature. This approach more accurately simulates the higher-pressure conditions typical of industrial carbon capture and utilization processes. The high CO_2_ adsorption capacity of sample A3 (with the biggest amino group load), compared to literature review studies (see Table 5), is largely attributed to raising the pressure from around 1 bar to 4 bar [25]. The higher CO_2_ pressure enhanced CO_2_ density and its interaction with amino groups on the aerogel surface, promoting efficient carbamate ion formation and enabling greater CO_2_ capture.

### 3.8. Nanocellulose Aerogel Lifespan

**Figure 8:**
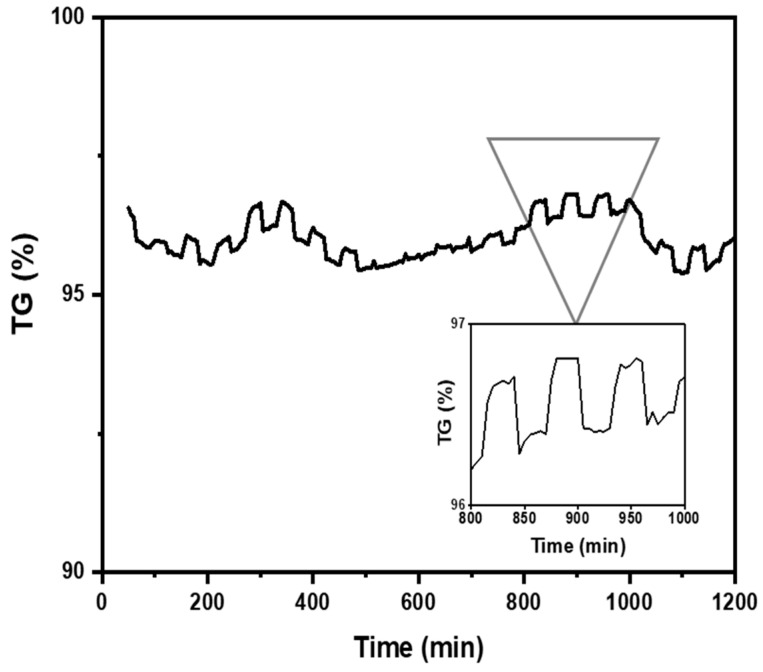
Nanocellulose aerogel (A3) adsorption/desorption cycles at 298 K and 1 bar determined by thermogravimetric analysis.

The study of CO_2_ adsorption/desorption cycles was conducted using the aerogel that exhibited the highest CO_2_ adsorption, specifically sample A3. Figure 8 shows that the CO_2_ desorption was fast and was complete after 30 min. A total of 95 wt.%. of the absorbed CO_2_ was desorbed during 20 adsorption/desorption cycles (reversible adsorption) measured at room temperature (298 K) in the thermobalance (TGA). It can be concluded that this number of cycles is likely higher since after 20 cycles, the aerogel continued to exhibit the same behavior as in the first cycle. Gebald et al. (2011) and Zhu et al. (2011) reported that CO_2_ desorption was fast and was complete after 30 min for a similar substrate. More than 85% of the CO_2_ was desorbed within 19 min at a packed bed temperature of below 80 °C [7,13,14].

**Figure 9:**
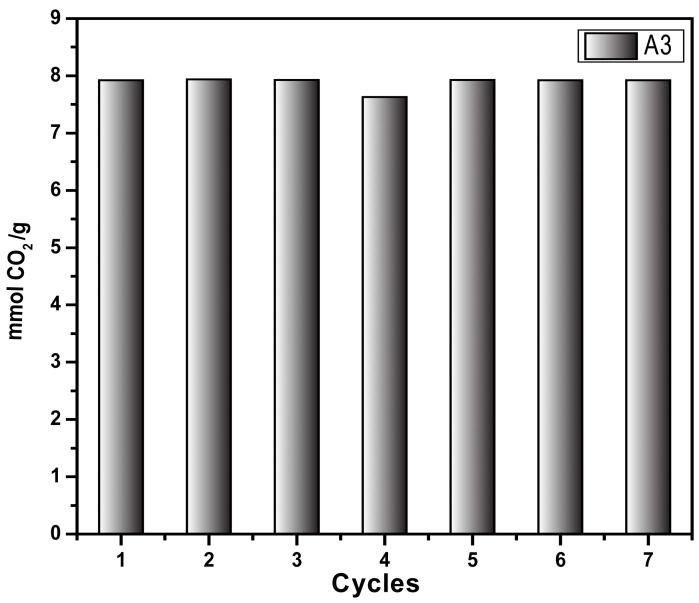
CO_2_ capture cycle of aerogel A3 at 318.15 K and 4 bar through the pressure decay technique.

Figure 9 shows the sorption/desorption test (pressure decay technique) using the same sample (A3) across seven cycles. The sample was regenerated by heating to 70 °C for 20 min at the end of each sorption test. The results prove that the sample retains the CO_2_ sorption capacity for up to seven cycles; this could likely be higher as the retained sample exhibited the same sorption capacity as in the first cycle, demonstrating the recyclability of the synthesized material. These results agree with results obtained from the adsorption/desorption cycles at 298 K determined by thermogravimetric analysis. Both techniques demonstrated the stability and reusability of the synthesized materials, which is one of the pre-requisites of the CO_2_-capture materials at the industrial scale. 

## 4. Conclusions

This study highlights the potential of DAMO-modified nanocellulose aerogels for scalable CO_2_ capture and storage applications. The predominant chemisorption mechanism, characterized by the interaction between CO_2_ molecules and amino groups to form covalent bonds, was found to be highly effective for CO_2_ adsorption. A significant increase in CO_2_ adsorption was observed with a slight increase in DAMO loading (4.62, 9.24, and 13.87 mmol of DAMO, respectively), ranging from 3.17 to 7.86 mmol CO_2_ g^−1^ at 298 K (25 °C) and 4 bar. The use of the decay pressure technique allowed the study to examine the effect of pressure on CO_2_ adsorption at pressures higher than atmospheric conditions, thus simulating real industrial conditions more closely.

The nanocellulose aerogels demonstrated excellent thermal stability at up to 240 °C and consistent performance across adsorption/desorption cycles, desorbing 95% of the adsorbed CO_2_ over 20 cycles at room temperature. This stability and reusability underscore their practicality for repeated use in industrial CO_2_ capture processes. These results support the feasibility of utilizing these biodegradable materials in scalable CO_2_ capture technologies, making them promising candidates for sustainable and efficient carbon capture alternatives. 

## Figures and Tables

**Table 1:** Isotherm models.

Isotherm	Equation
BET	$\frac{X}{\left(1-x\right)V}=\frac{1}{cV}+\frac{c-1}{cV}x$
Langmuir	$\frac{1}{V}=\frac{1}{KVP}+\frac{1}{V}$
Freundlich	θ = KP^1/n^
Temkin	θ = A ln (B.P)

**Table 2:** Nanocellulose aerogel elemental analysis.

Sample	C wt.%	H wt.%	N wt.%	O wt.%
A0	39.25	5.68	0.07	55.0
A1	39.80	6.13	0.35	53.7
A2	39.77	6.40	1.55	52.8
A3	39.97	6.75	2.74	50.5
STD *	71.64	6.64	10.42	11.3

* Calibration standard, L-cysteine.

**Table 3:** Isotherms parameters.

Isotherms	Parameters	A1	A2	A3
BET	S (g/cm^3^ STP)	0.2216	0.1843	0.2037
	Y (g/cm^3^ STP)	–0.0044	–0.0034	–0.0036
	R	0.9980	0.9982	0.9981
	Qm (cm^3^/g STP)	4.6032	5.5292	4.997
Langmuir	S (g/cm^3^ STP)	0.1695	0.1341	0.1576
	Y (g/cm^3^ STP)	–1.2550	–0.3500	–1.0360
	R	0.9988	0.9997	0.9987
	Qm (cm^3^/g STP)	5.8963	7.4543	6.3445
Freundlich	S	6.8946	6.5043	7.4617
	R	0.7788	0.8586	0.7864
	Qm (cm^3^/g STP)	2.7227	3.2204	3.1092
Temkin	S (mmHg^−1^)	268.6050	94.1404	441.948
	R	0.7887	0.8860	0.8080
	Qm kJ mol^−1^ (cm^3^ g^−1^ STP)	1.2021	0.8736	1.1657

**Table 4:** Nanocellulose aerogels: BET Area, Qm ^a^ and X0 ^b^.

Sample	BET Area (m^2^ g^−1^)	Qm (cm^3^ g^−1^)	X0 (mmol g^−1^)
A0	94.00	–	–
A1	154.00	4.60	0.21
A2	154.80	5.52	0.25
A3	154.30	4.99	0.22

^a^ CO_2_ amount absorbed under standard temperature and pressure conditions (cm^3^ g^−1^); ^b^ CO_2_ amount adsorbed under standard temperature and pressure conditions (mmol g^−1^).

**Table 5:** CO_2_ adsorption capacity of various cellulosic materials.

Cellulosic Materials	Functionalization	CO_2_ Adsorption Capacity (mmol g^−1^)	Reference
CNC and CNFs	AEAPDMS	2.36	[7]
CNFs	DAMO	2.16	[8]
CNFs	APS	1.91	[9]
CNFs	APMDS	1.02	[10]
CNC	AEAPMDS	2.63	[11]
CNC	AEAPMDS	1.59	[12]
CNFs	Phthalimide	5.20	[13]
CNFs	DAMO	7.86	This work

## Data Availability

The original contributions presented in this study are included in the article/Supplementary Material. Further inquiries can be directed to the corresponding author.

## Supplementary Materials

The following supporting information can be downloaded at: https://www.mdpi.com/article/10.3390/ma18020243/s1, Figure S1: Adsorption isotherm A1, Figure S2: Adsorption isotherm A1 in BET coordinates, Figure S3: Adsorption isotherm A1 in Langmuir coordinates, Figure S4: Adsorption isotherm A1 in Freundlich coordinates, Figure S5: Adsorption isotherm A1 in Temkin coordinates, Figure S6: Adsorption isotherm A2, Figure S7: Adsorption isotherm A2 in BET coordinates, Figure S8: Adsorption isotherm A2 in Langmuir coordinates, Figure S9: Adsorption isotherm A2 in Freundlich coordinates, Figure S10: Adsorption isotherm A2 in Temkin coordinates, Figure S11: Adsorption isotherm A3, Figure S12: Adsorption isotherm A3 in BET coordinates, Figure S13: Adsorption isotherm A3 in Langmuir coordinates, Figure S14: Adsorption isotherm A3 in Freundlich coordinates, Figure S15: Adsorption isotherm A3 in Temkin coordinates, Figure S16: Adsorption isotherm A1, A2 and A3; Table S1: Adsorption isotherm data A1, Table S2: BET report A1, Table S3: Langmuir Report A1, Table S4: Freundlich Report A1, Table S5: Temkin Report A1, Table S6: Adsorption isotherm data A2, Table S7: BET report A2, Table S8: Langmuir Report A2, Table S9: Freundlich Report A2, Table S10: Temkin Report A2, Table S11: Adsorption isotherm data A3, Table S12: BET report A3, Table S13: Langmuir Report A3, Table S14: Freundlich Report A3, Table S15: Temkin Report A3.
