# Supplementary material for: Stable Reusability of Nanocellulose Aerogels with Amino Group Modification in Adsorption/Desorption Cycles for CO2 Capture
Source: Materials (Basel). 2025 Jan 8;18(2):243. doi: 10.3390/ma18020243 (PMC11766845; doi:10.3390/ma18020243)
Supplement: Supplementary file 1 [file materials-18-00243-s001.zip › materials-3339761-supplementary.pdf]

# Stable Reusability of Nanocellulose Aerogels with Amino Group Modification in Adsorption/Desorption Cycles for $CO_2$ Capture

F Valdebenito *[1,2], C Albornoz [1], V Rivera [1], E Elgueta [1,2], M Nisar [2,5], S. Lira [4], O Valerio [3], A Narváez [2,5], CP Quezada[1], R Muñoz [1,2], L Azócar [1,2,6] and F Sandoval [1]

[1]Departamento de Química Ambiental, Universidad Católica de la Santísima Concepción, Concepción, Chile

[2]Centro de Energía, Universidad Católica de la Santísima Concepción, Concepción, Chile

[3]Departamento de ingeniería química, Universidad de Concepción, Concepción, Chile

[4]Center for Sustainability Research, Universidad Andres Bello, Santiago, Chile

[5]Departamento de ingeniería eléctrica, Universidad Católica de la Santísima Concepción, Concepción, Chile

[6]Centro de Investigación de Polímeros Avanzados, CIPA, Concepción, Chile.

*Corresponding author: fvaldebenitoi@ucsc.cl

# Supporting Information

## Content:

| | |
|---|---|
| Pages | 25 |
| Figures | 16 |
| Tables | 15 |

Detailed description of the different isotherm models (BET, Langmuir, Freundlich Temkin) used in this work to fit the experimental data (PDF)

Table SI-1. ADSORPTION ISOTHERM DATA A1

A1 AEROGEL 8-6-2023 - Adsorption

| Relative Pressure (P/Po) | Quantity Adsorbed (cm³/g STP) |
|---|---|
| 0,000892131 | 1,363326111 |
| 0,000999378 | 1,557560573 |
| 0,001121314 | 1,753216674 |
| 0,001200067 | 1,869039605 |
| 0,001279706 | 1,994748686 |
| 0,001355806 | 2,103279535 |
| 0,001457212 | 2,293225862 |
| 0,00154216 | 2,414713013 |
| 0,00159596 | 2,519743868 |
| 0,001638612 | 2,59883188 |
| 0,001722675 | 2,726011314 |
| 0,001782315 | 2,717131092 |
| 0,005589756 | 3,842707891 |
| 0,005628513 | 3,99316377 |
| 0,005657537 | 4,084730022 |
| 0,005692401 | 4,18963973 |
| 0,005734698 | 4,316316992 |
| 0,005791684 | 4,397152431 |
| 0,005844422 | 4,388776951 |
| 0,010124209 | 5,41958118 |
| 0,010150401 | 5,566146156 |
| 0,010185619 | 5,703120633 |
| 0,010205794 | 5,786309429 |
| 0,020522552 | 6,269745786 |
| 0,030289803 | 6,295215704 |
| 0,061291042 | 6,376559048 |
| 0,077721581 | 6,415643173 |
| 0,092839673 | 6,493226445 |
| 0,123678268 | 6,602686738 |
| 0,247758283 | 6,449986595 |
| 0,311253357 | 6,341751596 |
| 0,374389568 | 6,286836271 |
| 0,438305825 | 6,167968878 |
| 0,50133406 | 6,060999094 |
| 0,562461933 | 5,945867268 |

| | |
|---|---|
| 0,623436215 | 5,773803916 |
| 0,684868662 | 5,639115996 |
| 0,745803592 | 5,429047567 |
| 0,86824116 | 5,172033762 |
| 0,898975332 | 5,044400376 |
| 0,929475323 | 4,939681576 |
| 0,990886247 | 4,712587089 |

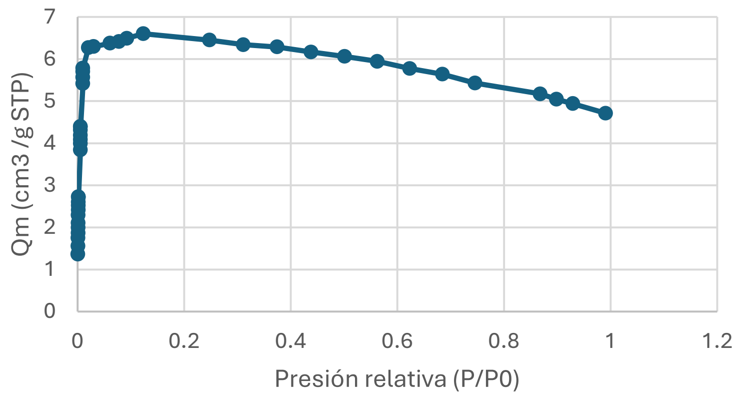


Figure SI-1. ADSORPTION ISOTHERM A1

Table SI-2. BET Report A1

BET Report

| | |
|---|---|
| BET Surface Area: | 21.0253 ± 0.7886 $m^2/g$ |
| Slope: | 0.221671 ± 0.008072 $g/cm^3$ STP |
| Y-Intercept: | -0.004431 ± 0.001113 $g/cm^3$ STP |
| C: | -49.023099 |
| Qm: | 4,6032 $cm^3/g$ STP |
| Correlation Coefficient: | 0.9980169 |
| Molecular Cross-Sectional Area: | 0.1700 $nm^2$ |

BET Surface Area Plot

A1 AEROGEL 8-6-2023 Not Fitted

| Relative Pressure (P/Po) | 1/[Q(Po/P - 1)] | Relative Pressure (P/Po) | 1/[Q(Po/P - 1)] |
|---|---|---|---|
| 0,061291042 | 0,010239521 | | |
| 0,077721581 | 0,013135281 | | |
| 0,092839673 | 0,01576119 | | |
| 0,123678268 | 0,021375147 | | |
| 0,247758283 | 0,051063662 | | |

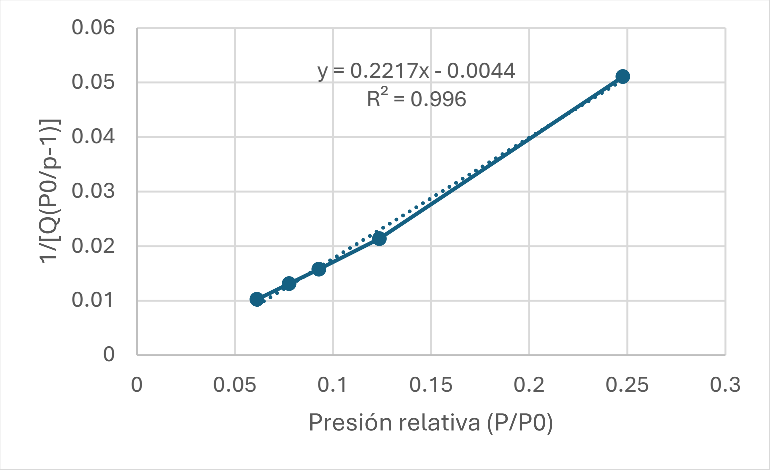


Figure SI-2. ADSORPTION ISOTHERM A1 IN BET COORDINATES

Table SI-3. Langmuir Report A1

| Langmuir Report | |
|---|---|
| Langmuir Surface Area: | 26,9316 ± 0,3930 m²/g |
| Slope: | 0,169597 ± 0,002475 g/cm³ STP |
| Y-Intercept: | -1,255 ± 0,635 mmHg·g/cm³ STP |
| b: | -0,135137 1/mmHg |
| Qm: | 5,8963 cm³/g STP |
| Correlation Coefficient: | 0.998831 |
| Molecular Cross-Sectional Area: | 0.1700 nm² |

Langmuir Surface Area Plot

| A1 AEROGEL 8-6-2023 | | Not Fitted | |
|---|---|---|---|
| Pressure (mmHg) | P/Q (mmHg·g/cm³ STP) | Pressure (mmHg) | P/Q (mmHg·g/cm³ STP) |

| | |
|---|---|
| 15,59713936 | 2,487682897 |
| 23,02025032 | 3,656784994 |
| 46,58119202 | 7,305067147 |
| 59,06840134 | 9,206933699 |
| 70,55815125 | 10,86642393 |
| 93,9954834 | 14,23594472 |
| 188,2962952 | 29,19328473 |
| 236,5525513 | 37,3008226 |
| 284,5360718 | 45,2590237 |
| 333,1124268 | 54,00682677 |
| 381,0138855 | 62,86321439 |
| 427,4710693 | 71,89381297 |
| 473,8115234 | 82,0622817 |

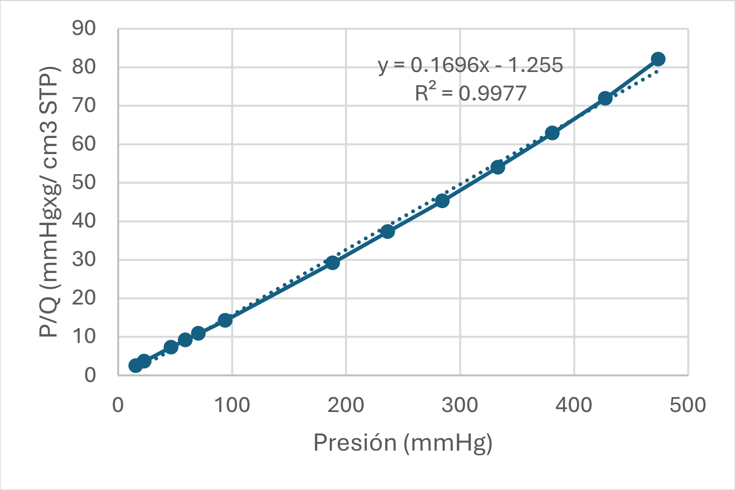


Figure SI-3. ADSORPTION ISOTHERM A1 IN LANGMUIR COORDINATES

Table SI-4. Freundlich Report A1

Freundlich Tabular Report

| | |
|---|---|
| Qm·C: | 2.7227 ± 0.0828 $cm^3/g$ STP |
| m: | 6.8946 ± 0.8778 |
| Correlation Coefficient: | 0.778878 |

Freundlich Plot

A1 AEROGEL 8-6-2023

| log(P) | log(Q) |
|---|---|
| -0,1687578 | 0,13459975 |
| -0,1194567 | 0,19244495 |
| -0,0694593 | 0,24383559 |
| -0,03998075 | 0,2716185 |
| -0,01207612 | 0,29988819 |
| 0,01301099 | 0,322897 |
| 0,04433638 | 0,36044683 |
| 0,06894305 | 0,38286552 |
| 0,08383572 | 0,4013564 |
| 0,0952896 | 0,41477819 |
| 0,11701685 | 0,43552765 |
| 0,1317981 | 0,43411059 |
| 0,62820641 | 0,58463737 |
| 0,63120727 | 0,60131712 |
| 0,63344097 | 0,61116336 |
| 0,63610908 | 0,62217668 |
| 0,63932413 | 0,63511333 |
| 0,64361842 | 0,64317152 |
| 0,64755519 | 0,64234351 |
| 0,8861747 | 0,73396573 |
| 0,8872968 | 0,74555461 |
| 0,88880104 | 0,75611256 |
| 0,88966039 | 0,76240165 |
| 1,19304495 | 0,79724993 |
| 1,36211004 | 0,79901062 |
| 1,6682106 | 0,80458639 |
| 1,77135522 | 0,8072402 |
| 1,84854719 | 0,81246055 |
| 1,97310699 | 0,81972069 |
| 2,27484178 | 0,80955881 |
| 2,37392764 | 0,80220923 |
| 2,45413733 | 0,79843215 |
| 2,52259083 | 0,79014217 |
| 2,5809408 | 0,78254422 |
| 2,63090673 | 0,77421521 |
| 2,67560562 | 0,76146203 |
| 2,71642089 | 0,75121103 |
| 2,75343806 | 0,73472365 |

| | |
|---|---|
| 2,81945396 | 0,71366135 |
| 2,83456137 | 0,70280955 |
| 2,84905146 | 0,69369895 |
| 2,87683739 | 0,67325939 |

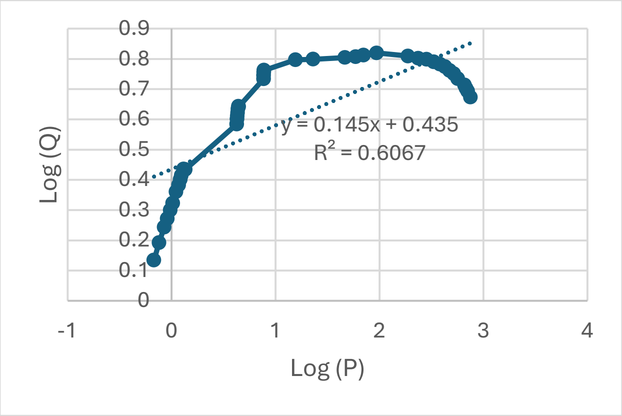


Figure SI-4. ADSORPTION ISOTHERM A1 IN FREUNDLICH COORDINATES

Table SI-5. Temkin Report A1

| Temkin Tabular Report | |
|---|---|
| q·alpha/Qm: | 1.202170 ± 0.148123 kJ/mol·(cm³/g STP) |
| A: | 268.6050 ± 223.6366 1/mmHg |
| Correlation Coefficient: | 0.788781 |

Temkin Plot

A1 AEROGEL 8-6-2023

| ln(P) | Quantity Adsorbed (cm³/g STP) |
|---|---|
| -0,3885792 | 1,36332611 |
| -0,27505921 | 1,55756057 |
| -0,15993596 | 1,75321667 |
| -0,09205908 | 1,86903961 |
| -0,0278063 | 1,99474869 |
| 0,02995891 | 2,10327953 |

| | |
|---|---|
| 0,10208828 | 2,29322586 |
| 0,15874723 | 2,41471301 |
| 0,19303889 | 2,51974387 |
| 0,21941241 | 2,59883188 |
| 0,26944125 | 2,72601131 |
| 0,30347634 | 2,71713109 |
| 1,44649872 | 3,84270789 |
| 1,45340845 | 3,99316377 |
| 1,45855175 | 4,08473002 |
| 1,46469529 | 4,18963973 |
| 1,4720982 | 4,31631699 |
| 1,48198618 | 4,39715243 |
| 1,49105092 | 4,38877695 |
| 2,04049265 | 5,41958118 |
| 2,04307639 | 5,56614616 |
| 2,04654002 | 5,70312063 |
| 2,04851875 | 5,78630943 |
| 2,74708752 | 6,26974579 |
| 3,13637428 | 6,2952157 |
| 3,84119685 | 6,37655905 |
| 4,07869612 | 6,41564317 |
| 4,25643721 | 6,49322644 |
| 4,54324673 | 6,60268674 |
| 5,23801676 | 6,4499866 |
| 5,46617039 | 6,3417516 |
| 5,65086004 | 6,28683627 |
| 5,80848005 | 6,16796888 |
| 5,94283582 | 6,06099909 |
| 6,05788661 | 5,94586727 |
| 6,16080961 | 5,77380392 |
| 6,25479024 | 5,639116 |
| 6,34001953 | 5,42904757 |
| 6,49203266 | 5,17203376 |
| 6,52681875 | 5,04440038 |
| 6,56018341 | 4,93968158 |
| 6,6241629 | 4,71258709 |

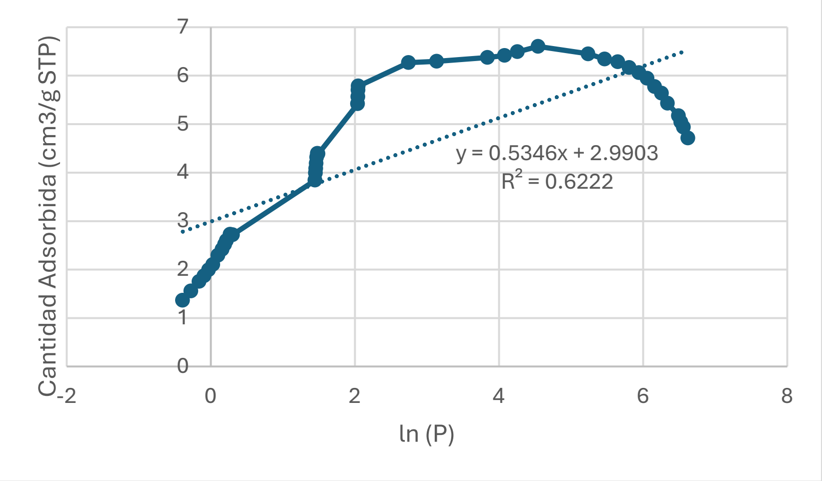


Figure SI-5. ADSORPTION ISOTHERM A1 IN TEMKIN COORDINATES

Table SI-6. ADSORPTION ISOTHERM A2 DATA

A2 AEROGEL 8-6-2023 - Adsorption

| Relative Pressure (P/Po) | Quantity Adsorbed (cm³/g STP) |
|---|---|
| 0,001063974 | 1,926874279 |
| 0,001208031 | 2,155389105 |
| 0,001322003 | 2,329347941 |
| 0,00140819 | 2,462026619 |
| 0,001544461 | 2,696631802 |
| 0,001623038 | 2,777088611 |
| 0,001699668 | 2,884236435 |
| 0,001780192 | 3,000216155 |
| 0,001827975 | 3,098122303 |
| 0,001897526 | 3,183409375 |
| 0,001954158 | 3,299335875 |
| 0,00202601 | 3,355356357 |
| 0,004723994 | 4,118057707 |
| 0,004767353 | 4,237523233 |
| 0,004797792 | 4,328857891 |
| 0,00483655 | 4,387788041 |
| 0,004878493 | 4,477079504 |
| 0,004954415 | 4,425299546 |
| 0,009563552 | 5,787516397 |
| 0,009597177 | 5,947113747 |
| 0,009637528 | 6,07880611 |
| 0,00967133 | 6,19471901 |

| | |
|---|---|
| 0,009730439 | 6,324324422 |
| 0,020156391 | 7,296950413 |
| 0,030078659 | 7,415590463 |
| 0,061479523 | 7,498544991 |
| 0,077327282 | 7,611386202 |
| 0,092680389 | 7,74692606 |
| 0,123840031 | 7,78474978 |
| 0,247638281 | 7,726188217 |
| 0,310960468 | 7,706115012 |
| 0,37436937 | 7,683794808 |
| 0,438432553 | 7,566173398 |
| 0,501197253 | 7,480241792 |
| 0,562735186 | 7,467076734 |
| 0,623419551 | 7,397411046 |
| 0,685007437 | 7,347234342 |
| 0,745732438 | 7,199973814 |
| 0,86807781 | 6,968040788 |
| 0,898993884 | 6,94340972 |
| 0,929719543 | 6,878338163 |
| 0,990964468 | 6,731049251 |

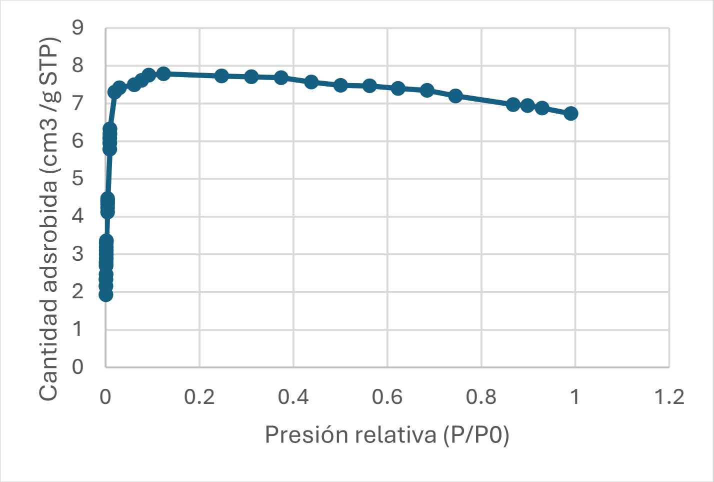


Figure SI-6. ADSORPTION ISOTHERM A2

Table SI-7. BET Report A2

BET Report

BET Surface Area: 25.2545 ± 0.8818 $m^2/g$

| | |
|---|---|
| Slope: | 0.184353 ± 0.006256 g/cm³ STP |
| Y-Intercept: | -0.003494 ± 0.000862 g/cm³ STP |
| C: | -51.768397 |
| Qm: | 5,5292 cm³/g STP |
| Correlation Coefficient: | 0.9982771 |
| Molecular Cross-Sectional Area: | 0.1700 nm² |

| A2 AEROGEL 8-6-2023 | | Not Fitted | |
|---|---|---|---|
| Relative Pressure (P/Po) | 1/[Q(Po/P - 1)] | Relative Pressure (P/Po) | 1/[Q(Po/P - 1)] |
| 0,061479523 | 0,008735942 | | |
| 0,077327282 | 0,011010862 | | |
| 0,092680389 | 0,013185547 | | |
| 0,123840031 | 0,018156536 | | |
| 0,247638281 | 0,04260159 | | |

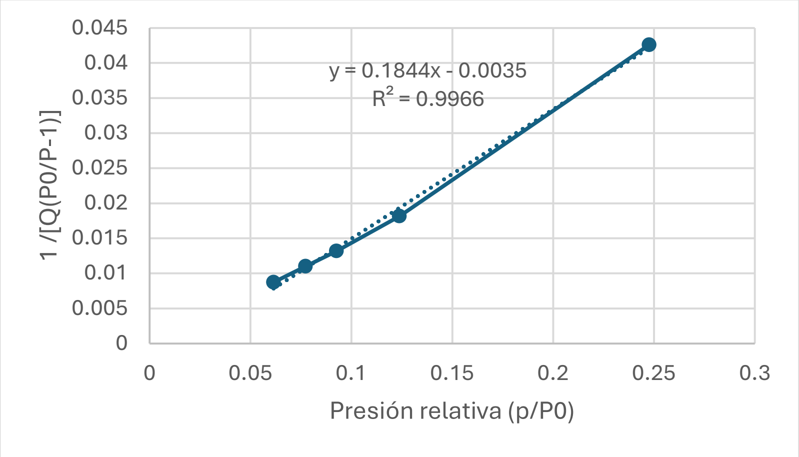


Figure SI-7. ADSORPTION ISOTHERM A2 IN BET COORDINATES

Table SI-8. Langmuir Report A2

Langmuir Report

| | |
|---|---|
| Langmuir Surface Area: | 34,0477 ± 0,2228 m²/g |
| Slope: | 0,134151 ± 0,000878 g/cm³ STP |
| Y-Intercept: | -0,350 ± 0,225 mmHg·g/cm³ STP |
| b: | -0,383784 1/mmHg |
| Qm: | 7,4543 cm³/g STP |
| Correlation Coefficient: | 0.999765 |

| | |
|---|---|
| Molecular Cross-Sectional Area: | 0.1700 nm² |

Langmuir Surface Area Plot

| A2 AEROGEL 8-6-2023 | | Not Fitted | |
|---|---|---|---|
| Pressure (mmHg) | P/Q (mmHg·g/cm³ STP) | Pressure (mmHg) | P/Q (mmHg·g/cm³ STP) |
| 15,31885719 | 2,099350595 | | |
| 22,8597908 | 3,082666298 | | |
| 46,72443771 | 6,231133876 | | |
| 58,76873398 | 7,721160432 | | |
| 70,43709564 | 9,092263834 | | |
| 94,11842346 | 12,09010259 | | |
| 188,2050934 | 24,35937206 | | |
| 236,3299561 | 30,66784699 | | |
| 284,5207214 | 37,02867249 | | |
| 333,2087402 | 44,03926829 | | |
| 380,9099121 | 50,92213898 | | |
| 427,6787415 | 57,27525733 | | |
| 473,7988586 | 64,04928098 | | |

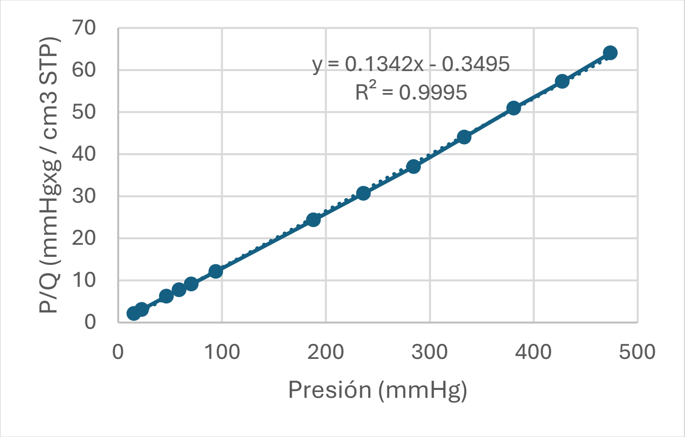


Figure SI-8. ADSORPTION ISOTHERM A2 IN LANGMUIR COORDINATES

Table SI-9. Freundlich Report A2

Freundlich Tabular Report

| | |
|---|---|
| Qm·C: | 3.2204 ± 0.0768 $cm^3/g$ STP |
| m: | 6.5043 ± 0.6139 |
| Correlation Coefficient: | 0.858657 |

Freundlich Plot

A2 AEROGEL 8-6-2023

| log(P) | log(Q) |
|---|---|
| -0,0922555 | 0,28485338 |
| -0,03710821 | 0,33352568 |
| 0,00204612 | 0,36723437 |
| 0,02947485 | 0,39129274 |
| 0,06959049 | 0,43082165 |
| 0,09114222 | 0,44358974 |
| 0,11117765 | 0,46003052 |
| 0,13128032 | 0,47715255 |
| 0,14278381 | 0,49109856 |
| 0,15900131 | 0,50289249 |
| 0,17177324 | 0,51842653 |
| 0,18745515 | 0,52573865 |
| 0,5551229 | 0,61469243 |
| 0,55909087 | 0,62711209 |
| 0,56185502 | 0,63637333 |
| 0,56534926 | 0,64224564 |
| 0,56909925 | 0,65099481 |
| 0,575806 | 0,64594267 |
| 0,86143282 | 0,76249223 |
| 0,86295711 | 0,77430625 |
| 0,86477923 | 0,78381829 |
| 0,86629979 | 0,79202161 |
| 0,86894605 | 0,80101414 |
| 1,18522637 | 0,8631414 |
| 1,35907225 | 0,87014574 |
| 1,66954408 | 0,874977 |
| 1,76914634 | 0,88146376 |
| 1,84780144 | 0,88912941 |
| 1,97367464 | 0,89124466 |
| 2,27463137 | 0,88796528 |
| 2,37351877 | 0,88683549 |
| 2,4541139 | 0,88557576 |

| | |
|---|---|
| 2,52271638 | 0,87887629 |
| 2,58082227 | 0,87391564 |
| 2,63111766 | 0,87315061 |
| 2,67559401 | 0,86907975 |
| 2,71650888 | 0,86612389 |
| 2,75339663 | 0,85733092 |
| 2,81937225 | 0,84311068 |
| 2,83457033 | 0,84157279 |
| 2,84916555 | 0,83748352 |
| 2,87687167 | 0,82808277 |

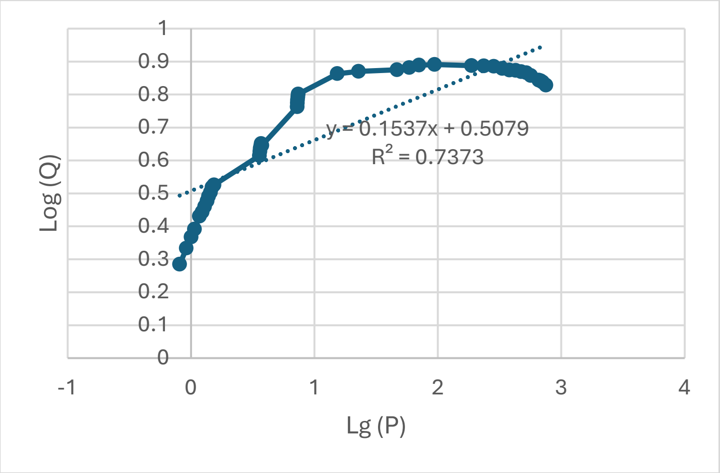


Figure SI-9. ADSORPTION ISOTHERM A2 IN FREUNDLICH COORDINATES

Temkin Tabular Report

| | |
|---|---|
| q·alpha/Qm: | 0.873691 ± 0.072292 kJ/mol·(cm³/g STP) |
| A: | 94.1404 ± 46.0775 1/mmHg |
| Correlation Coefficient: | 0.886013 |

Table SI-10. Temkin Report A2

Temkin Plot

A3 AEROGEL 7-6-2023

| ln(P) | Quantity Adsorbed (cm³/g STP) |
|---|---|
| -0,447828534 | 1,50595474 |
| -0,308002517 | 1,759583486 |
| -0,138543981 | 2,089506069 |
| -0,089408121 | 2,221788543 |
| -0,020091629 | 2,359220747 |
| 0,031002595 | 2,494223976 |
| 0,091343404 | 2,680652791 |
| 0,132234151 | 2,824663685 |
| 0,165153165 | 2,923678887 |
| 0,196028483 | 3,02977257 |
| 0,244371074 | 3,183328175 |
| 0,287965929 | 3,223978629 |
| 1,038672096 | 4,001742153 |
| 1,050596649 | 4,109359115 |
| 1,060193485 | 4,214491544 |
| 1,072510188 | 4,27986156 |
| 1,549050108 | 5,014004735 |
| 1,550048828 | 5,094652748 |
| 1,557019151 | 5,224362188 |
| 1,57611228 | 5,215743632 |
| 2,096261344 | 5,98015985 |
| 2,09806176 | 6,04308754 |
| 2,100320078 | 6,202773293 |
| 2,756844604 | 6,475458514 |
| 3,137699663 | 6,603161896 |
| 3,843818103 | 6,674072474 |
| 4,076920695 | 6,782663179 |
| 4,250447006 | 6,780156683 |
| 4,542983471 | 7,036342097 |
| 5,239036241 | 6,958253552 |
| 5,465966854 | 6,808896974 |
| 5,651048141 | 6,728156367 |
| 5,807785746 | 6,652385255 |
| 5,94285312 | 6,492604755 |
| 6,058089841 | 6,378341272 |
| 6,161220005 | 6,200712971 |
| 6,254796455 | 6,168285288 |

| | |
|---|---|
| 6,339969227 | 6,014719347 |
| 6,492287182 | 5,711260645 |
| 6,526630504 | 5,562970835 |
| 6,560115238 | 5,474287392 |
| 6,624318982 | 5,269364125 |

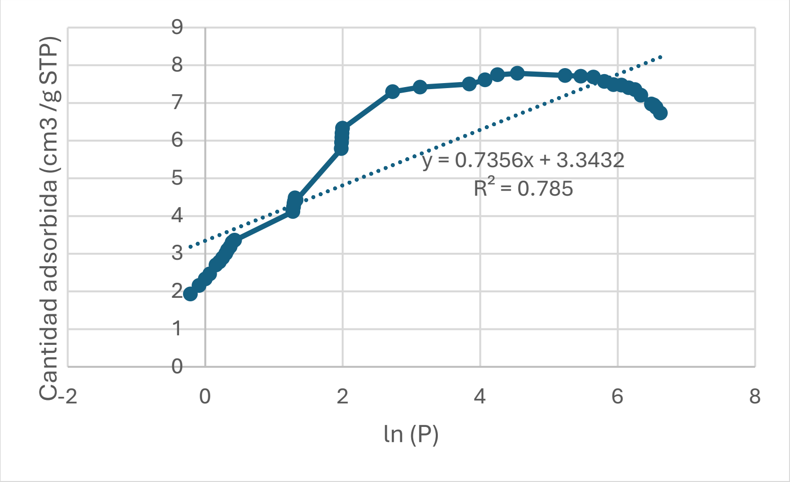


Figure SI-10. ADSORPTION ISOTHERM A2 IN TEMKIN COORDINATES

Table SI-11. ADSORPTION ISOTHERM A3 DATA

A3 AEROGEL 7-6-2023 - Adsorption

| Relative Pressure (P/Po) | Quantity Adsorbed (cm³/g STP) |
|---|---|
| 0,000840808 | 1,50595474 |
| 0,000966991 | 1,759583486 |
| 0,001145559 | 2,089506069 |
| 0,001203253 | 2,221788543 |
| 0,001289617 | 2,359220747 |
| 0,001357221 | 2,494223976 |
| 0,001441638 | 2,680652791 |
| 0,00150181 | 2,824663685 |
| 0,001552071 | 2,923678887 |
| 0,001600739 | 3,02977257 |
| 0,001680024 | 3,183328175 |
| 0,001754884 | 3,223978629 |
| 0,003717714 | 4,001742153 |
| 0,003762311 | 4,109359115 |
| 0,003798591 | 4,214491544 |
| 0,003845667 | 4,27986156 |

| | |
|---|---|
| 0,006193417 | 5,014004735 |
| 0,006199611 | 5,094652748 |
| 0,00624297 | 5,224362188 |
| 0,006363313 | 5,215743632 |
| 0,010704864 | 5,98015985 |
| 0,010724154 | 6,04308754 |
| 0,0107484 | 6,202773293 |
| 0,020723772 | 6,475458514 |
| 0,030329975 | 6,603161896 |
| 0,061451912 | 6,674072474 |
| 0,077583715 | 6,782663179 |
| 0,092285206 | 6,780156683 |
| 0,123645712 | 7,036342097 |
| 0,248010997 | 6,958253552 |
| 0,311190013 | 6,808896974 |
| 0,374459999 | 6,728156367 |
| 0,438001613 | 6,652385255 |
| 0,501342733 | 6,492604755 |
| 0,562576254 | 6,378341272 |
| 0,623692121 | 6,200712971 |
| 0,684872918 | 6,168285288 |
| 0,745761671 | 6,014719347 |
| 0,868462171 | 5,711260645 |
| 0,89880612 | 5,562970835 |
| 0,929411958 | 5,474287392 |
| 0,991040922 | 5,269364125 |

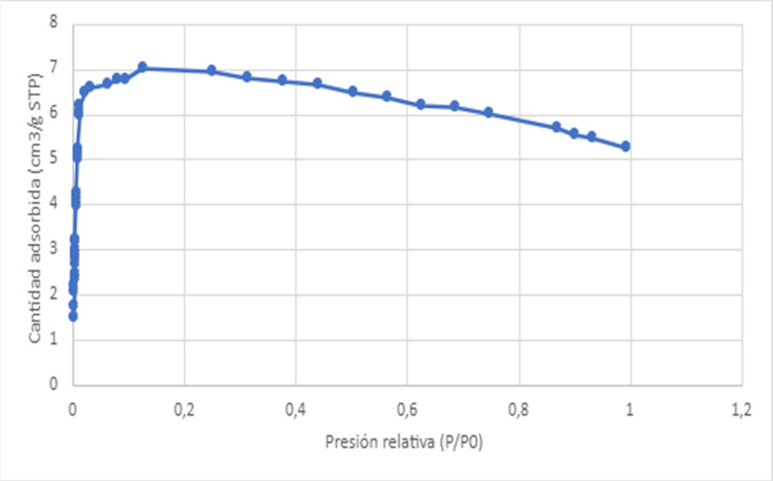


Figure SI-11. ADSORPTION ISOTHERM A3

Table SI-12. BET Report A3

BET Report

| | | | |
|---|---|---|---|
| BET Surface Area: | 22.8268 ± 0.8259 m²/g | | |
| Slope: | 0.203733 ± 0.007172 g/cm³ STP | | |
| Y-Intercept: | -0.003638 ± 0.000989 g/cm³ STP | | |
| C: | -54.999087 | | |
| Qm: | 4,9976 cm³/g STP | | |
| Correlation Coefficient: | 0.9981463 | | |
| Molecular Cross-Sectional Area: | 0.1700 nm² | | |
| A3 AEROGEL 7-6-2023 | | Not Fitted | |
| Relative Pressure (P/Po) | 1/[Q(Po/P - 1)] | Relative Pressure (P/Po) | 1/[Q(Po/P - 1)] |
| 0,061451912 | 0,009810428 | | |
| 0,077583715 | 0,012400619 | | |
| 0,092285206 | 0,014994878 | | |
| 0,123645712 | 0,020051727 | | |
| 0,248010997 | 0,047397911 | | |

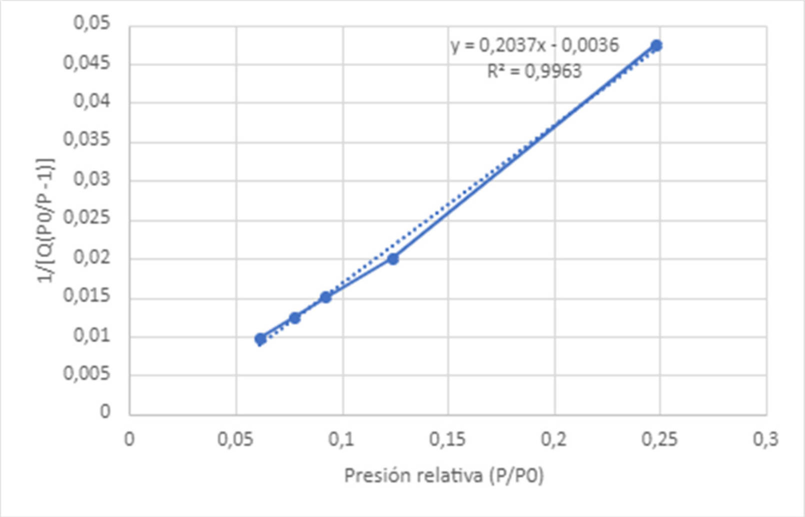

Figure SI-12. ADSORPTION ISOTHERM A3 IN BET COORDINATES

Table SI-13. Langmuir Report A3

Langmuir Report

| | | | |
|---|---|---|---|
| Langmuir Surface Area: | 28,9786 ± 0,4361 m²/g | | |
| Slope: | 0,157617 ± 0,002372 g/cm³ STP | | |
| Y-Intercept: | -1,036 ± 0,609 mmHg·g/cm³ STP | | |
| b: | -0,152151 1/mmHg | | |
| Qm: | 6,3445 cm³/g STP | | |
| Correlation Coefficient: | 0.998757 | | |
| Molecular Cross-Sectional Area: | 0.1700 nm² | | |
| A3 AEROGEL 7-6-2023 | | Not Fitted | |
| Pressure (mmHg) | P/Q (mmHg·g/cm³ STP) | Pressure (mmHg) | P/Q (mmHg·g/cm³ STP) |
| 15,75003906 | 2,432270506 | | |
| 23,05078125 | 3,490870225 | | |
| 46,70345306 | 6,997744368 | | |
| 58,96362305 | 8,693284849 | | |
| 70,1367569 | 10,34441535 | | |
| 93,97074127 | 13,35505579 | | |
| 188,4883575 | 27,08845778 | | |
| 236,5044098 | 34,73461424 | | |

| | |
|---|---|
| 284,5895996 | 42,29830344 |
| 332,8812256 | 50,03937878 |
| 381,0204773 | 58,685303 |
| 427,5579529 | 67,03278088 |
| 474,006012 | 76,4437919 |

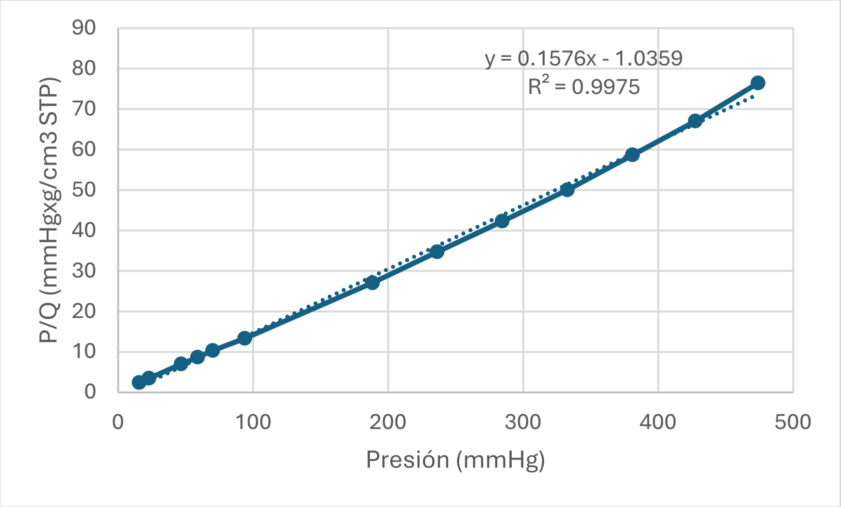


Figure SI-13. ADSORPTION ISOTHERM A3 IN LANGMUIR COORDINATES

Table SI-14. Freundlich Report A3

| | |
|---|---|
| Freundlich Tabular Report | |
| Qm·C: | 3.1092 ± 0.0854 cm³/g STP |
| m: | 7.4617 ± 0.9322 |
| Correlation Coefficient: | 0.784644 |

Freundlich Plot

A3 AEROGEL 7-6-2023

| log(P) | log(Q) |
|---|---|
| -0,194489461 | 0,17781192 |
| -0,133763793 | 0,245409877 |

| | |
|---|---|
| -0,060168886 | 0,320042725 |
| -0,038829454 | 0,346702723 |
| -0,008725713 | 0,372768579 |
| 0,013464256 | 0,39693545 |
| 0,039669936 | 0,428240566 |
| 0,057428562 | 0,450966747 |
| 0,071725108 | 0,465929672 |
| 0,085134088 | 0,481410029 |
| 0,106129009 | 0,502881413 |
| 0,125062014 | 0,508392154 |
| 0,45108956 | 0,602249102 |
| 0,456268327 | 0,613774096 |
| 0,46043618 | 0,624745187 |
| 0,465785256 | 0,631429721 |
| 0,672743914 | 0,70018474 |
| 0,673178067 | 0,707114588 |
| 0,676204826 | 0,718033278 |
| 0,684496866 | 0,717316236 |
| 0,910394734 | 0,776712793 |
| 0,911176645 | 0,781258885 |
| 0,91215742 | 0,792585908 |
| 1,197282399 | 0,811270525 |
| 1,362685649 | 0,819751945 |
| 1,669348992 | 0,824390918 |
| 1,770584161 | 0,831400251 |
| 1,84594568 | 0,83123973 |
| 1,972992653 | 0,847346946 |
| 2,27528453 | 0,84250025 |
| 2,373839243 | 0,833076763 |
| 2,454219025 | 0,827896076 |
| 2,522289302 | 0,822977392 |
| 2,580948317 | 0,812418965 |
| 2,630994989 | 0,804707752 |
| 2,67578385 | 0,792441628 |
| 2,716423586 | 0,790164452 |
| 2,753413651 | 0,779215368 |
| 2,819564498 | 0,756731981 |
| 2,834479613 | 0,745306783 |
| 2,849021848 | 0,738327593 |

| | |
|---|---|
| 2,87690518 | 0,72175821 |

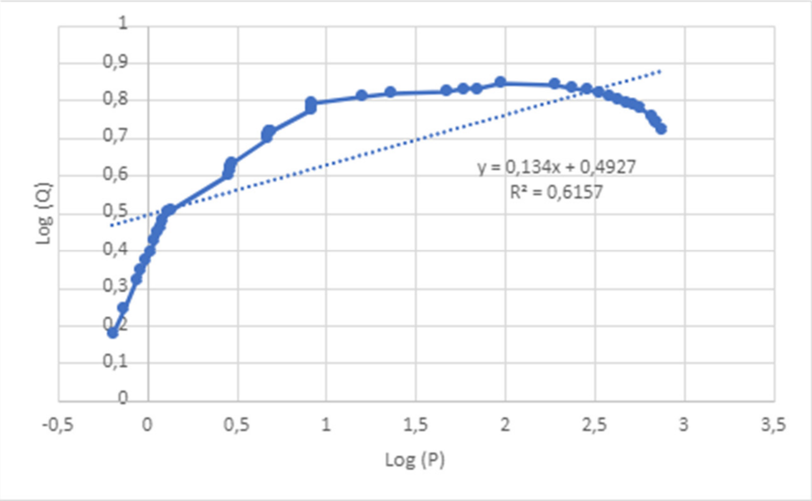


Figure SI-14. ADSORPTION ISOTHERM A3 IN FREUNDLICH COORDINATES

Table SI-15. Temkin Report A3

Temkin Tabular Report

| | |
|---|---|
| q·alpha/Qm: | 1.165734 ± 0.134397 kJ/mol·(cm³/g STP) |
| A: | 441.9475 ± 365.2043 1/mmHg |
| Correlation Coefficient: | 0.808012 |

Temkin Plot

A3 AEROGEL 7-6-2023

| ln(P) | Quantity Adsorbed (cm³/g STP) |
|---|---|
| -0,447828534 | 1,50595474 |
| -0,308002517 | 1,759583486 |
| -0,138543981 | 2,089506069 |
| -0,089408121 | 2,221788543 |

| | |
|---|---|
| -0,020091629 | 2,359220747 |
| 0,031002595 | 2,494223976 |
| 0,091343404 | 2,680652791 |
| 0,132234151 | 2,824663685 |
| 0,165153165 | 2,923678887 |
| 0,196028483 | 3,02977257 |
| 0,244371074 | 3,183328175 |
| 0,287965929 | 3,223978629 |
| 1,038672096 | 4,001742153 |
| 1,050596649 | 4,109359115 |
| 1,060193485 | 4,214491544 |
| 1,072510188 | 4,27986156 |
| 1,549050108 | 5,014004735 |
| 1,550048828 | 5,094652748 |
| 1,557019151 | 5,224362188 |
| 1,57611228 | 5,215743632 |
| 2,096261344 | 5,98015985 |
| 2,09806176 | 6,04308754 |
| 2,100320078 | 6,202773293 |
| 2,756844604 | 6,475458514 |
| 3,137699663 | 6,603161896 |
| 3,843818103 | 6,674072474 |
| 4,076920695 | 6,782663179 |
| 4,250447006 | 6,780156683 |
| 4,542983471 | 7,036342097 |
| 5,239036241 | 6,958253552 |
| 5,465966854 | 6,808896974 |
| 5,651048141 | 6,728156367 |
| 5,807785746 | 6,652385255 |
| 5,94285312 | 6,492604755 |
| 6,058089841 | 6,378341272 |
| 6,161220005 | 6,200712971 |
| 6,254796455 | 6,168285288 |
| 6,339969227 | 6,014719347 |
| 6,492287182 | 5,711260645 |
| 6,526630504 | 5,562970835 |
| 6,560115238 | 5,474287392 |
| 6,624318982 | 5,269364125 |

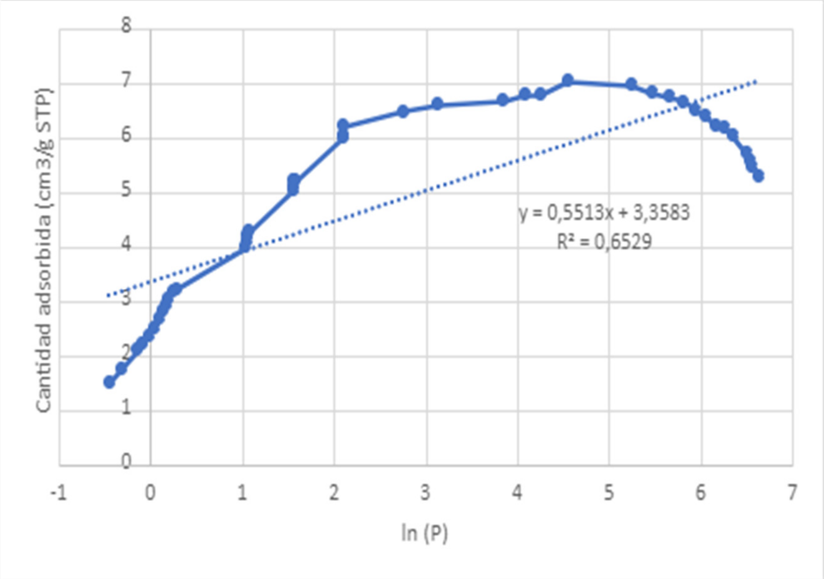

Figure SI-15. ADSORPTION ISOTHERM A3 IN TEMKIN COORDINATES

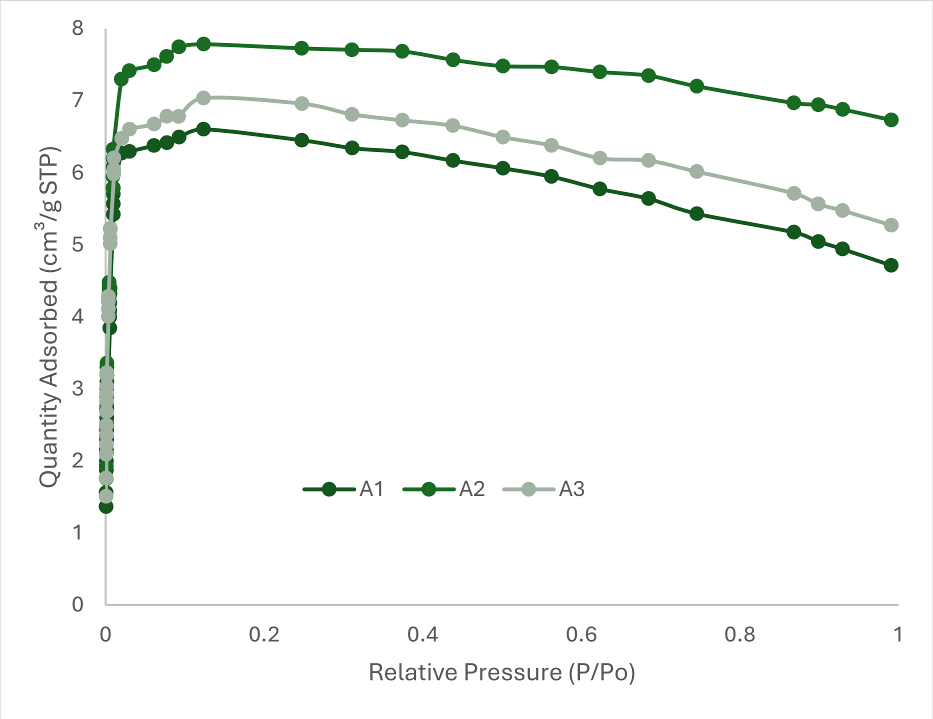

Figure SI-16. ADSORPTION ISOTHERM A1, A2 AND A3
